# Parental Autonomy Support and Pathological Internet Use among Chinese Undergraduate Students: Gratitude Moderated the Mediating Effect of Filial Piety

**DOI:** 10.3390/ijerph19052644

**Published:** 2022-02-24

**Authors:** Chunhua Ma, Yongfeng Ma, Xiaoyu Lan

**Affiliations:** 1 College of Educational Science and Technology, Northwest Minzu University, Lanzhou 730030, China; mch@xbmu.edu.cn (C.M.); mayongfeng@xbmu.edu.cn (Y.M.); 2 Promenta Research Center, Department of Psychology, University of Oslo, 0373 Oslo, Norway

**Keywords:** pathological internet use, parental autonomy support, filial piety, gratitude, Chinese undergraduate students

## Abstract

Applying an integrated theoretical model consisting of the socioecological theory, the self-determination theory, and the broaden-and-build theory, the present study tested a moderated mediation model of parental autonomy support, filial piety, and gratitude to study how these factors are jointly related to pathological Internet use (PIU) in Chinese undergraduate students. A total of 1054 Chinese undergraduate students (*M* age = 20.35, *SD* = 1.00, 34.7% females) aged between 16 and 24 years participated in this study. They were instructed to complete self-reported questionnaires on parental autonomy support, filial piety, gratitude, and PIU. The results showed that parental autonomy support was negatively associated with PIU, and filial piety partially mediated this relation. Specifically, parental autonomy support was positively related to filial piety, which, in turn, was negatively associated with PIU. In addition, gratitude moderated the first path of the indirect relation and the direct relation of this mediation effect. To be specific, undergraduate students with higher gratitude showed high filial piety and low PIU, in the context of low parental autonomy support, than those with lower gratitude. Taken together, the current study contributes to extant research by highlighting the vital role of parental autonomy support in mitigating undergraduate students’ PIU and illustrating how filial piety explains the underlying mechanism of this association. This study also provides novel insights into intervention or prevention programs by demonstrating that gratitude alleviates the adverse effect of low parental autonomy support on students’ PIU.

## 1. Introduction

With the popularization of the Internet, the number of Internet users in China is multiplying. According to a recent national survey, as of June 2021, Chinese Internet users accounted for 1.011 billion people, an increase of 21.75 million from December 2020 [1]. The Internet has indeed brought significant convenience to our daily lives, but it also has had several negative impacts, such as the growth of pathological Internet use (PIU). PIU refers to a series of problematic symptoms caused by excessive or poorly controlled use of the Internet, including changes in mood, failure to fulfill obligations, and interpersonal conflicts [2,3]. PIU negatively affects people’s psychosocial functions, especially for undergraduate students [3,4,5]. Since undergraduate students lack the physical supervision of their parents and their lives are relatively independent, they can extensively indulge in online games and social interaction software for hours a day [5,6,7]. Many empirical studies have specifically pointed out that PIU has a negative effect on the mental and physical health, social behavior, and academic performance of Chinese undergraduate students [4,8,9]. Given that PIU adversely affects various functions of undergraduate students, it is imperative to study the correlates of PIU in this population.

Although a burgeoning body of studies has investigated the correlates of PIU among undergraduate students, researchers have not yet produced a rigorous and comprehensive investigation of how contextual and individual variables are related to PIU. To fill this research gap, in the current study, we employed a socioecological theory [10], as an overarching framework to understand the complex associations of variables of interest situated in multiple levels affecting PIU. According to this theory, individuals are embedded in various layers of the system, and their development occurs due to the influence of multiple factors between contextual (e.g., parental autonomy support) and individual variables (e.g., filial piety and gratitude). In accordance with the current study, several empirical studies have used this theoretical framework to identify specific correlates that influence addictive behaviors [11,12,13]. Despite this, previous studies usually focused on a narrow range of factors, either contextual or individual, preventing researchers and educators from gaining a thorough and holistic understanding of this phenomenon. This is particularly striking given that PIU involves a complex underlying mechanism and multiple levels of processes.

To further extend this line of research, we integrated the umbrella theory (i.e., socioecological theory) with other relevant, but broad, theories (i.e., self-determination theory and broaden-and-build theory; see elaborations in the following sections) to enhance specificity when crafting the variables located in different layers. Using such an approach would reduce the redundancy among distinct theories and leverage their strengths in a bid to gain a more comprehensive understanding of the informative correlates of PIU [14]. Theoretically, integrating different perspectives will advance existing scholarship and trigger insightful theoretical reflections on PIU. Practically, this integrative approach provides important insights into designing specific and systematic intervention or prevention programs on undergraduate students’ PIU by combining the strengths of each theory.

In the following elements of the introduction, we conduct a literature review organized by each focused variable, and we generate the research hypotheses leading the current study.

### 1.1. Parental Autonomy Support and Pathological Internet Use

According to self-determination theory (SDT) [15,16], autonomy is one of the three fundamental psychological needs (autonomy, competence, and relatedness) which creates a sense of initiative and ownership of one’s actions. Nevertheless, the satisfaction of autonomy requires supportive conditions to be robust [17]. Within parenting, autonomy support involves understanding children’s feelings, allowing children to express their opinions freely, and encouraging children to choose their own actions voluntarily [15,18]. Consistent with these theoretical claims, recent studies have shown that parental autonomy support can promote their children’s adaptive behavioral outcomes, psychological thriving, and academic performance, such as (but not limited to) better social functioning [19,20], health behavior [21], high life satisfaction [22], academic success and school engagement [23]. For undergraduate students, autonomy is considered a core developmental component, and autonomy support from others is critical to adaptive development at this stage [7,24]. Although undergraduate students live far away from their parents and spend more time with their teachers and peers on campus, they still tend to regard their parents as one of their primary sources of social support for crucial matters [25,26]. It is reasonable that parental autonomy support can meet the autonomy need of undergraduate students, which is vital for their successful transition to adulthood [19,27].

Previous empirical studies have shown that parenting practices are an essential buffer against students’ PIU [28,29]. This is because the Internet can meet their belonging needs that are not met in the real world. Despite these findings, research on the association of parental autonomy support with PIU is relatively limited. Since parental autonomy support can positively affect students’ internal motivation by enabling them to regulate their emotions and behaviors appropriately, it is possible that parental autonomy support may serve as a protective factor for PIU. In addition, we focus on parental autonomy support of Chinese undergraduate students because of its cultural salience in autonomy research. Previous research on autonomy has mainly focused on groups in Western cultural contexts. In contrast, exploration of this line of research in the Eastern cultural context has been relatively scarce. Some researchers posit that collectivism attaches great importance to social harmony and interdependence, and therefore the positive association between autonomy support and psychosocial functions may not be so salient [30]. However, with the rapid economic development and socio-cultural transformations seen in contemporary China, autonomy support has become more valued among the young generations [31]. It is necessary to expand prior research by studying the association of parental autonomy support with PIU among Chinese undergraduate students living in collectivistic cultural contexts emphasizing interdependence.

### 1.2. Filial Piety as a Mediator

Filial piety, anchored in traditional Confucian ethics, has been defined as a set of normative notions and moral underpinnings regarding how children should behave towards their parents [32,33]. Individuals with high filial piety often treat their elderly parents with tremendous love and respect, a high degree of compliance, and an unswerving commitment to caring for them [33]. Generations of Chinese, even in contemporary Chinese societies [34,35], have emphasized the importance of filial piety, which plays a critical role in family heritage and maintaining the social order. Although previous studies have not verified the mediating role of filial piety in the association between parental autonomy support and PIU, relevant studies have shown that filial piety plays a mediating role between parental autonomy support and adolescents’ psychosocial functions [22,36]. This can be explained by SDT [15], suggesting that parental autonomy support could fulfill individuals’ need for autonomy. This positive parent–child relationship helps individuals to accept and internalize the concept of filial piety, and individuals with high filial piety tend to adjust their behaviors to repay their parents. Therefore, in the present study, we propose that filial piety can mediate the negative relationship between parental autonomy support and PIU.

First, parental autonomy support may positively promote undergraduate students’ filial piety. Parents are essential transmitters of undergraduate students’ social values, including filial piety. Parents pass social and cultural values on to their children through family socialization [37,38], and the way in which parents care for and interact with their children will affect the development of filial piety [30,39]. Parental autonomy support can establish a good parent–child relationship, which helps undergraduate students realize the importance of respecting and caring for their parents, so that undergraduate students are willing to perform their filial duties [22,33].

Second, filial piety may negatively impact undergraduate students’ PIU. In traditional Chinese culture, filial piety includes not only love and respect for parents but also the fulfillment of a series of family responsibilities [32,33,40]. Therefore, we propose that undergraduate students with filial piety may strive for excellence and try to regulate their behavior so that it does not violate the wishes of their parents and the dignity of their families. Moreover, as the basis of the parent–child relationship, filial piety comes from the family, but its function is not limited to the family. Research has shown that people with filial piety have better interpersonal relationships and can more easily obtain social support [37,41]. In this perspective, undergraduates with filial piety may feel more warmth and support in daily life instead of seeking support from the Internet. Therefore, filial piety may be negatively associated with PIU.

### 1.3. Gratitude as a Moderator

Socioecological theory [10] emphasizes the impact of the interaction between contextual and individual variables on the psychosocial outcomes of individuals. Therefore, when parental autonomy support affects PIU via filial piety, it may depend on other individual variables, such as gratitude. Gratitude has been defined as a life orientation towards noticing and appreciating the positive experiences and achievements in the world [42,43]. Grateful individuals are likely to feel more frequent and intense positive emotions, have a more positive perspective of their surroundings, and adopt optimistic coping strategies, consequently achieving a better physical condition and mental integration [43,44,45]. Previous research has found that gratitude plays a moderating role in contextual and individual behavioral development [46,47]. In accordance with this empirical finding, we postulate that gratitude may moderate the process of parental autonomy support that affects PIU through filial piety.

According to Wood et al. [48], individuals with high gratitude have more active coping strategies and achieve their goals through long-term persistence. Even in unfavorable situations, individuals with high gratitude can face difficulties and achieve their goals by adjusting their behavior. For example, Lo et al. have found that gratitude plays a moderating role between the contextual variables and problem behaviors, and it can alleviate the impact of negative parenting styles on children’s problem behaviors [47]. In line with this finding, we propose that gratitude may moderate the association of parental autonomy support with PIU. At the same level of parental autonomy support, undergraduate students with higher levels of gratitude are more likely to adopt positive coping strategies, enabling them to regulate their behavior adequately, thereby reducing the levels of PIU.

In addition, gratitude may moderate the relationship between parental autonomy support and filial piety. According to the broaden-and-build theory [49], positive emotions, such as gratitude, can broaden the scope of individual cognition and enhance cognitive flexibility, which is conducive to the construction of individual psychological and social resources and the adjustment of cognition and behavior. Individuals with high gratitude tend to have lower gratitude thresholds, explain the behavior of others in a positive way, and are more likely to perceive that others are supportive and available, thus rewarding their benefactors [42,43,50]. Therefore, we propose that, under the same level of parental autonomy support, undergraduate students with high gratitude are more sensitive to the warmth and support of their parents, and they are therefore more willing to repay their parents.

### 1.4. The Present Study

**Figure 1:**
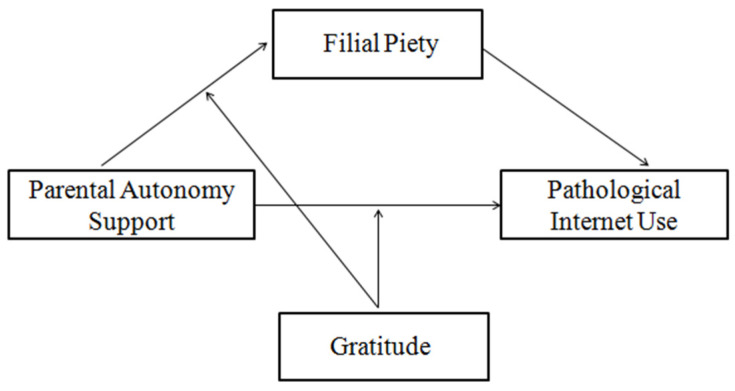
A hypothesized model of the study associations.

Applying an integrated theoretical model consisting of socioecological theory, SDT, and the broaden-and-build theory, the present study examines the association between parental autonomy support and PIU in Chinese undergraduate students. Furthermore, it explores the mediating role of filial piety and the moderating role of gratitude in order to determine “when” and “how” parental autonomy support is negatively related to undergraduate students’ PIU (see Figure 1). The hypotheses of the present study are as follows: (1) parental autonomy support would be negatively associated with PIU, (2) filial piety would partially mediate the relationship between autonomy support and PIU; to be specific, parental autonomy support would be positively associated with filial piety, which, in turn, would be negatively related to PIU and (3) gratitude might moderate the first path of the indirect relation and the direct relation of this mediation effect. In addition, previous studies have suggested that pathological Internet users are more likely to be older and male [3,51,52]. Therefore, we considered undergraduate students’ age and gender as covariates when testing the aforementioned research hypotheses.

## 2. Methods

### 2.1. Participants and Procedures

The present study is based on a non-experimental, cross-sectional design. Before data collection, the research protocols and procedures were carefully reviewed and approved by the Research Ethics Committee of the College of Educational Science and Technology, Northwest Minzu University. Following this approval, the authors contacted the responsible deans and professors of two universities in Lanzhou, Gansu Province (a Province located in Northwest mainland China), for their consent. Subsequently, the authors delivered a questionnaire QR code via WeChat (a widely used social media software application in China) to undergraduate students during school hours, and invited them to complete this web-based, well-established survey. Upon scanning this QR code using a mobile phone, a detailed explanation about research purposes, participants’ rights, and ethical guarantees was highlighted, and students were asked for digital consent before the actual investigation began. Participation in the current study was anonymous and entirely voluntary, and students could withdraw from this study any time they wanted without external pressure. After the data were collected, the responses remained highly confidential and would only be used for scientific purposes, with their identification information anonymized. All data collection and management procedures were strictly conducted following the Declaration of Helsinki and its later amendments.

A total of 1081 Chinese undergraduate students participated in the study, and 27 cases were excluded from the current study due to duplicate submission and being outliers (observed score was either above or below three *SDs*) in at least one of the variables of interest. A valid sample of 1054 students was therefore included for further analysis, with a 97.5% effective sample rate. Participants were 16–24 years old (*M* age = 20.35, *SD* = 1.00; 34.7% females). All four grades during undergraduate education in China were included, with 27.2% freshmen, 49.0% sophomores, 15.7% juniors, and 8.1% seniors. Although we did not collect the information concerning students’ courses they took in the college, two universities where the data were collected were specified to focus on hard science and social science education, respectively. Therefore, we presumed that their professional backgrounds were, at least to some degree, balanced. In terms of their family demographic information, 26.1% of participants reported being a single child in their families, while the remaining percent varied by different numbers of siblings. Most of their fathers (56.8%) and mothers (50.8%) had completed high school or college education. In addition, their family residences were well-balanced between rural (53.3%) and urban regions (46.7%).

### 2.2. Measures

#### 2.2.1. Parental Autonomy Support

We applied the parental autonomy support questionnaire to assess parental autonomy support [53]. This questionnaire includes eight items. One item was, “*My parents allow me to plan what I want to do*”. This was scored on a 5-point Likert scale ranging from 1 (completely disagree) to 5 (completely agree). The average score was computed in this study. The questionnaire demonstrated a good internal consistency among Chinese college students [20]. Cronbach’s alpha was 0.93 in this study.

#### 2.2.2. Filial Piety

We applied the filial piety scale to assess filial piety [33]. This scale includes ten items. One item was, “*I am grateful to my parents for raising me*”. This was scored on a 5-point Likert scale ranging from 1 (strongly disagree) to 5 (strongly agree). The average score was computed, with higher scores indicating higher filial piety beliefs. Previous research reported that the questionnaire has good reliability among Chinese samples [33,54]. Cronbach’s alpha was 0.93 in this study.

#### 2.2.3. Gratitude

We applied the gratitude questionnaire-6 to assess gratitude [42]. This questionnaire consists of six items. One item example is, “*I have so much in life to be thankful for*”. This was scored on a 7-point Likert scale ranging from 0 (completely disagree) to 6 (completely agree). Among these six items, the third item (“*Looking around the world, there is nothing I am grateful for*”) was deleted due to a low correlation with other items in the scale, suggesting that participants may have difficulties understanding this item. Therefore, this item was omitted from further analysis. A higher average score for all remaining items indicates higher levels of gratitude in this study. Previous research reported that the questionnaire has good reliability among Chinese college students [55]. Cronbach’s alpha was 0.63 in this study.

#### 2.2.4. Pathological Internet Use

PIU was measured using a pathological Internet use questionnaire [3]. This questionnaire includes 13 items. One item example is, “*I have been told I spend too much time online*”. In the current study, we used a 5-point Likert scale in the current study ranging from 1 (never) to 5 (always), rather than the dichotomous (yes/no) answer format in the original questionnaire, in order to obtain a more detailed picture of the PIU. A higher average score for all items indicates higher levels of PIU. Cronbach’s alpha was 0.91 in this study.

#### 2.2.5. Data Analysis Plan

Data analyses were performed using SPSS 25.0 (IBM Corp., Armonk, NY, USA) and the macro program PROCESS developed by Hayes [56]. First, we calculated the descriptive statistics (*M* and *SD)* and Pearson correlations for all variables. Second, we followed MacKinnon’s procedure to test the mediation model of filial piety [57]. Third, the analysis of the moderated mediation model was conducted. In this analysis, the bootstrap method was used to test (conditional) indirect effects, and 5000 samples were repeatedly drawn to estimate the 95% confidence intervals (*CIs*) of the various indirect effects. If the 95% *CI* did not include zero, the (conditional) indirect effects were considered statistically significant. Finally, we performed a simple slope analysis to probe the interaction effects.

## 3. Results

### 3.1. Preliminary Analyses

Table 1 shows the descriptive statistics and correlations of all variables. The results found that PAS and filial piety were negatively associated with PIU. PAS was positively associated with filial piety. Gratitude was positively associated with PIU. In terms of covariates, we did not find significant correlations between age and the study variables, but males reported significantly higher levels of PAS, filial piety, and gratitude than females. In addition, the values of skewness and kurtosis were in the range of −1 to 1, indicating that the variables did not violate the normality assumption [58].

### 3.2. Test for Mediation

The mediation model was tested according to MacKinnon’s mediation analysis method [57]. After controlling for age and gender, all variables were standardized, and Model 4 in the macro program PROCESS for SPSS was used to verify the mediating effect of filial piety. The results showed that parental autonomy support was negatively associated with PIU, and the total effect was significant (*b* = −0.69, *p* < 0.001). Hypothesis 1 was, therefore, supported.

**Figure 2:**
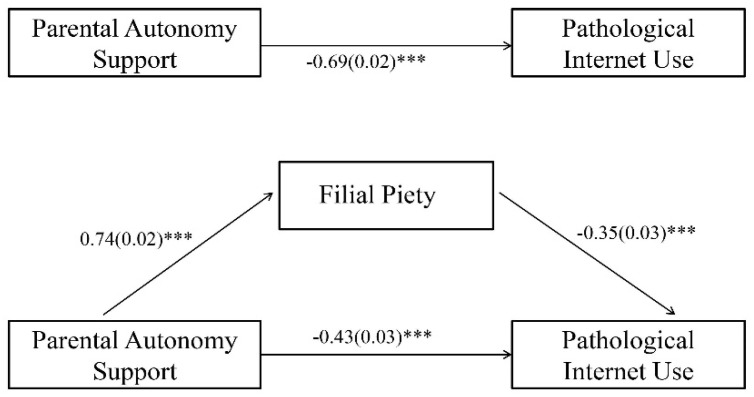
A mediation model. Note. N = 1054. Path coefficients are shown close to the lines, whereas standard errors are displayed inside the brackets. *** *p* < 0.001.

Subsequently, PAS was positively associated with filial piety (*b* = 0.74, *p* < 0.001), and filial piety was negatively associated with PIU (*b* = −0.35, *p* < 0.001). After filial piety was entered the regression equation, PAS was negatively associated with PIU, and the direct effect remained significant (*b* = −0.43, *p* < 0.001) (see Table 2). Bootstrap testing showed the following results: the indirect effect = −0.26, Boot*SE* = 0.03, 95% *CI* = [−0.32, −0.20]. In this regard, the mediation model established showed that filial piety partially mediated the relationship between PAS and PIU (see Figure 2). As such, Hypothesis 2 was corroborated.

### 3.3. Test for Moderated Mediation

We then examined whether gratitude moderated the first path of the indirect relation and the direct relation of the mediation effect. We used Model 8 in the macro program PROCESS for SPSS to examine the moderated mediation model. The results showed that PAS was negatively associated with PIU (*b* = −0.39, *p* < 0.001), gratitude was positively associated with PIU (*b* = 0.06, *p* < 0.05), and the interaction between PAS and gratitude was positively associated with PIU (*b* = −0.18, *p* < 0.05). In addition, PAS was positively associated with filial piety (*b* = 0.56, *p* < 0.001), gratitude was positively associated with filial piety (*b* = 0.24, *p* < 0.001), and the interaction between PAS and gratitude was negatively associated with filial piety (*b* = −0.18, *p* < 0.05). This showed that gratitude not only moderated the association between PAS and PIU but also moderated the association between PAS and filial piety (see Table 3). In terms of covariates, the results showed that age was positively associated with PIU (*b* = 0.06, *p* < 0.01), and males had higher filial piety (*b* = −0.15, *p* < 0.001) and PIU (*b* = −0.12, *p* < 0.05) scores than females.

To understand how gratitude moderated the relationship between PAS and PIU, gratitude was divided into three levels according to 1 *SD* above the mean, the mean, and 1 *SD* below the mean. The mediating effect of filial piety and its bootstrap confidence interval at different gratitude levels are shown in Table 4, where the index = 0.03, Boot*SE* = 0.01, 95% *CI* = [0.01, 0.04]. In this regard, a moderated mediation model was established, and Hypothesis 3 was confirmed.

**Figure 3:**
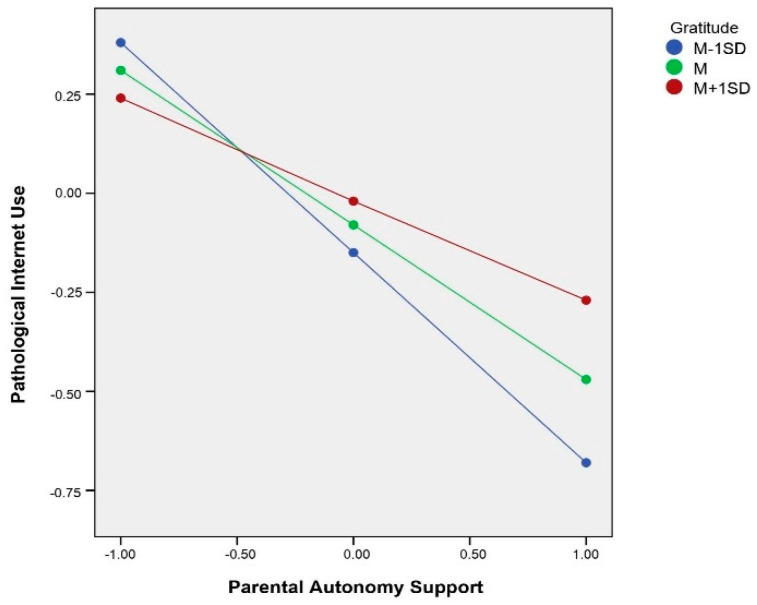
Interaction between parental autonomy support and gratitude in predicting pathological Internet use.

**Figure 4:**
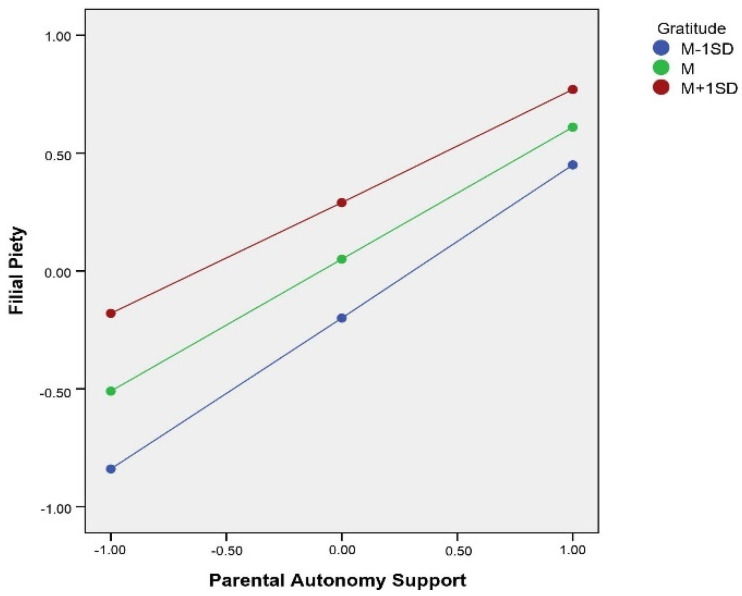
Interaction between parental autonomy support and gratitude in predicting filial piety.

Simple slope tests showed that the association between PAS and PIU was significantly negative at both higher levels of gratitude (*b* = −0.25, *p* < 0.001), and lower levels of gratitude (*b* = −0.53, *p* < 0.001). This indicated that, in the context of low levels of PAS, the impact on undergraduate students’ PIU was significantly alleviated by higher gratitude. In addition, the association between PAS and filial piety was significantly positive at both higher levels of gratitude (*b* = 0.48, *p* < 0.001), and lower levels of gratitude (*b* = 0.64, *p* < 0.001). This indicated that, in the context of low levels of PAS, higher gratitude significantly alleviated the negative impact of low levels of PAS on filial piety in undergraduate students (see Figure 3 and Figure 4). As a note, statistical power and interpretation of this moderated effect might be biased by methodological limitation, as the gratitude questionnaire employed showed a low internal consistency in this study. Thus, conclusions should be made bearing this limitation in mind.

## 4. Discussion

College life is a crucial period for individuals to develop autonomy and independence, and undergraduate students who live far from their parents face greater pressure and more challenges during this period of their lives [59]. Using the Internet may be an important way for these students to relieve pressure. Consequently, PIU has become a significant risk factor for their positive psychosocial functions. Based on an integrated theoretical model, this study examined the negative association between parental autonomy support and PIU in undergraduate students, and further investigated the mediating role of filial piety and the moderating role of gratitude in this association. The results showed that parental autonomy support was negatively associated with PIU, and filial piety partially mediated this relation. In addition, gratitude moderated the first path of the indirect relation and the direct relation of this mediation model.

The primary purpose of this study was to examine the association between parental autonomy support and PIU among Chinese undergraduate students. The results showed that parental autonomy support was negatively associated with PIU. The results supported Hypothesis 1 and were consistent with previous research findings, showing that parental autonomy support is related to the psychosocial adjustment of undergraduate students [19,27]. One possible explanation for these findings is that undergraduate students in early adulthood enjoy more freedom and autonomy than adolescents in general, and students who gain autonomy have a stronger sense of responsibility and an ability to resist risks [15,59]. During this period, although living far from their parents, students have the intrinsic motivation to regulate their behavior if they can obtain autonomy support from their parents, and they can resist the temptation of the Internet even without physical supervision [24,60]. Another possible explanation is that individuals who receive parental autonomy support have specific social skills and self-confidence [61]. Undergraduate students who receive autonomy support when they feel pressure may be more proactive in seeking help in reality, rather than using the Internet to relieve pressure. Finally, this result has also enriched the theoretical research of SDT [15,16], indicating that, in the context of collectivistic culture, parental autonomy support plays an essential role in combating individuals’ problem behaviors. This result is consistent with recent findings [31]. One possible interpretation for these results is that with the development of China’s economy and the intercultural exchange between Eastern and Western entities, autonomy has also become valued in contemporary Chinese society, especially by young generations, such as undergraduate students. Since undergraduate students face fierce social competition, autonomy and independence can motivate them to fight challenges and achieve success in social competition [62].

The second purpose of this study was to examine the mediating role of filial piety in the relationship between parental autonomy support and PIU. The results verified Hypothesis 2, showing that parental autonomy support was positively associated with filial piety, which, in turn, was negatively related to PIU. These results were consistent with research on adolescents showing that filial piety plays a mediating role between parental autonomy support and individual psychosocial development [36,37]. There are several possible interpretations of the findings. First, filial piety comes from the intergenerational transmission of family values, and parental autonomy support is considered an essential source of inculcating filial piety in undergraduate students [38,63]. Autonomy support from parents can make undergraduate students feel their parents respect and pay attention to their needs, thus generating a desire to repay their parents, which promotes the development of filial piety [33]. Second, filial piety, as the foundation of Chinese people’s thoughts and behaviors, can promote positive development functions in individuals and help them resist the risk of negative developments [22,41]. Undergraduate students with filial piety may apply good relations with their parents to their active interactions with others and thereby obtain supportive resources more easily [33,40]. Third, undergraduate students with high levels of filial piety may internalize their parents’ values related to education, have a strong sense of mission and family honor, and show obedience and commitment to their parents, thereby reducing the risk of PIU [22,41]. Therefore, the impact of parental autonomy support on PIU was achieved via the mediation of filial piety.

The third purpose of this study was to explore the moderating role of gratitude. The results verified Hypothesis 3, showing that gratitude moderated the mediation model. First, the results show that gratitude moderated the relationship between parental autonomy support and PIU. Further analysis shows that the role of gratitude was mainly reflected in unfavorable situations, where it could alleviate the negative impact of low parental autonomy support for undergraduate students’ PIU. This finding was consistent with previous research [48]. One possible reason for this finding is that when parental autonomy support is insufficient, undergraduate students with high levels of gratitude may have a higher capacity for self-control, which may suppress PIU as they persist and pursue their goals in reality. Under the same conditions, undergraduate students with low levels of gratitude may not effectively regulate their behavior, making it easier to seek a desire on the Internet, which will lead to the risk of PIU [64]. Second, gratitude also moderated the association between parental autonomy support and filial piety. This result is consistent with previous research finding that individuals with high levels of gratitude are more sensitive to others, making them more inclined to return gratitude to others [42,50]. The role of gratitude is still reflected in unfavorable situations. One possible reason for this is that undergraduate students with high levels of gratitude feel more positive emotions and fewer negative emotions, and are more likely to feel gratitude toward their parents. Another possible reason is that undergraduate students with high levels of gratitude may have positive regard for their parents. Even in the context of low parental autonomy support, they can still treat their parents with tolerance, and when they cannot return to their parents, they may feel guilt, shame, or feel they owe their parents [50,65]. Therefore, undergraduate students with high levels of gratitude have a higher level of filial piety than undergraduate students with low levels of gratitude. This difference was more evident in the context of insufficient parental autonomy support. In summary, gratitude buffers against the negative impact of unfavorable situations on filial piety and health behaviors.

## 5. Limitations

Although this study obtained important findings of the correlations of PIU in undergraduate students, it contains several limitations. One of the significant limitations of this study is related to the measurement of gratitude, in that the internal consistency of this scale was low. Future research should employ a more robust measurement technique to assess this construct and to attempt to replicate the current findings. Another limitation is the cross-sectional design of the present study, which restricted us from inferring causality of study associations. Future research could use longitudinal data to infer the directionality of variables of interest. In addition, the present study relies on self-reported questionnaires. Although our research meets the measurement standards, excessive reliance on a single method may lead to common method deviations. Future research should adopt multiple methods to study these variables. Finally, the current study only investigated a relatively limited number of the variables related to PIU, whereas other variables (e.g., autonomy support from other social agents, such as teachers, peers, and romantic partners; individual characteristics, such as grit and resilience) not considered in this study should be incorporated into future studies, to provide a more systematic understanding of the contextual and individual correlates of PIU.

## 6. Conclusions

Despite these limitations, the findings of the present research have important implications for college-based intervention or prevention programs. First, educators or counselors may consider holding structured presentations targeting parents during college registration or via web-based meetings when possible. Through this professional guidance, educators or counselors should emphasize the critical role of autonomy support in their children’s health outcomes, as well as in their career goals and well-being in general. Moreover, educators or counselors should supervise parents to take their children’s perspectives into account and to encourage their children to pursue personal interests instead of controlling and directly rejecting their children’s proposals. Alternatively, educators or counselors could also consider integrating these structured presentations with students’ general curriculum, motivating them to speak with their parents regarding the importance of autonomy support in the family. Nevertheless, in the context of families providing low autonomy support, educators or counselors should work on instilling or reinforcing their values of filial piety and gratitude. For instance, they may help students reminisce about unforgettable scenarios when their parents prudentially took care of them during childhood and adolescence. Induced by these retrospective memorials, professionals should motivate them to care for their aging parents and fulfill filial responsibility as a spiritual return. In addition, educators or counselors could induce gratitude through a few school activities, such as constructing a list of individuals about whom students feel grateful (gratitude lists), writing and delivering a letter to those they had not amply thanked (behavioral expressions of gratitude), and grateful contemplation [66].

To briefly summarize, the current study verifies the important role of parental autonomy support for undergraduate students’ PIU in the context of collectivism. It emphasizes that, when designing interventions to help undergraduate students overcome PIU, consideration should be given to cultivating and improving levels of filial piety and gratitude in undergraduate students.

## Figures and Tables

**Table 1:** Descriptive statistics and bivariate correlations of study variables.

Variable	*M*	*SD*	Range	Skewness	Kurtosis	1	2	3	4	5	6
1. PAS	3.49	0.85	1–5	−0.79	−0.23	-					
2. FP	3.45	0.85	1–5	−0.45	−0.37	0.75 **	-				
3. Gratitude	3.62	0.99	0–6	−0.19	−0.61	0.59 **	0.60 **	-			
4. PIU	2.74	0.76	1–5	0.42	−0.55	−0.68 **	−0.66 **	0.26 ***	-		
5. Age	20.35	1.00	16–24	-	-	0.02	0.01	0.03	0.05	-	
6. Gender ^a^	-	-	1–2	-	-	−0.10 **	−0.15 **	−0.14 **	0.04	0.01	-

*Note. N* = 1054. ^a^ coded as 1 = male, 2 = female. PAS = parental autonomy support; FP = filial piety; PIU = pathological Internet use. ** p < 0.01, *** *p* < 0.001.

**Table 2:** Mediating effect of filial piety.

	Model 1		Model 2		Model 3	
	PIU		FP		PIU	
Predictor	*b*	*t*	*b*	*t*	*b*	*t*
Gender ^a^	−0.06	−1.34	−0.15	−3.43 ***	−0.12	−2.55 *
Age	0.06	2.63 **	−0.01	−0.30	0.06	2.68 **
PAS	−0.69	−30.41 ***	0.74	36.32 ***	−0.43	−13.24 ***
FP					−0.35	−10.87 ***
*R* ^2^	0.47		0.57		0.52	
*F*	310.75 ***		457.24 ***		288.60 ***	

*Note. N* = 1054. ^a^ coded as 1 = male, 2 = female. PAS = parental autonomy support; FP = filial piety; PIU = pathological Internet use. * p < 0.05, ** p < 0.01, *** *p* < 0.001.

**Table 3:** Results of a moderated mediation model.

Outcome	Predictor	*R*	*R* ^2^	*F*	*b*	*t*	*LLCI*	*ULCI*
FP	Age	0.78	0.61	329.09 ***	−0.01	−0.39	−0.05	0.03
	Gender ^a^				−0.10	−2.42 *	−0.18	−0.02
	PAS				0.56	21.63 ***	0.51	0.61
	Gratitude				0.24	10.02 ***	0.19	0.29
	PAS × Gratitude				−0.08	−3.90 ***	−0.12	−0.04
PIU	Age	0.74	0.54	206.56 ***	0.05	2.37 *	0.01	0.09
	Gender ^a^				−0.12	−2.69 **	−0.21	−0.03
	PAS				−0.39	−11.50 ***	−0.46	−0.32
	FP				−0.35	−10.38 ***	−0.41	−0.28
	Gratitude				0.06	2.17 *	0.01	0.11
	PAS × Gratitude				0.14	6.09 ***	0.09	0.18

*Note. N* = 1054. ^a^ coded as 1 = male, 2 = female. PAS = parental autonomy support; FP = filial piety; PIU = pathological Internet use. * p < 0.05, ** p < 0.01, *** *p* < 0.001.

**Table 4:** The mediating effect of filial piety in the association of parental autonomy support and pathological Internet use at different levels of gratitude.

Gratitude	Conditional Direct (Indirect) Effect	*SE* (Boot*SE*)	*LLCI* (Boot*LLCI*)	*ULCI* (Boot*ULCI*)
*M-SD*	−0.53	0.04	−0.60	−0.46
	(−0.22)	(0.03)	(−0.28)	(−0.17)
*M*	−0.39	0.03	−0.46	−0.32
	(−0.20)	(0.03)	(−0.25)	(−0.14)
*M+SD*	−0.25	0.05	−0.34	−0.16
	(−0.17)	(0.03)	(−0.22)	−0.12)

*Note. N* = 1054.

## Data Availability

The data presented in this study are available on request from the corresponding author.
